# Influence of Jail Incarceration and Homelessness Patterns on Engagement in HIV Care and HIV Viral Suppression among New York City Adults Living with HIV/AIDS

**DOI:** 10.1371/journal.pone.0141912

**Published:** 2015-11-23

**Authors:** Sungwoo Lim, Denis Nash, Laura Hollod, Tiffany G. Harris, Mary Clare Lennon, Lorna E. Thorpe

**Affiliations:** 1 Bureau of Epidemiology Services, New York City Department of Health and Mental Hygiene, Queens, New York, United States of America; 2 CUNY School of Public Health, City University of New York, New York, New York, United States of America; 3 Monitoring and Evaluation, Corporate Contributions, Johnson & Johnson, New Brunswick, New Jersey, United States of America; 4 ICAP, Mailman School of Public Health, Columbia University, New York, New York, United States of America; 5 Bureau of Epidemiology Services, New York City Department of Health and Mental Hygiene, Queens, New York, United States of America; 6 The PhD program in Sociology, Graduate Center, City University of New York, New York, New York, United States of America; British Columbia Centre for Excellence in HIV/AIDS, CANADA

## Abstract

**Objectives:**

Both homelessness and incarceration are associated with housing instability, which in turn can disrupt continuity of HIV medical care. Yet, their impacts have not been systematically assessed among people living with HIV/AIDS (PLWHA).

**Methods:**

We studied a retrospective cohort of 1,698 New York City PLWHA with both jail incarceration and homelessness during 2001–05 to evaluate whether frequent transitions between jail incarceration and homelessness were associated with a lower likelihood of continuity of HIV care during a subsequent one-year follow-up period. Using matched jail, single-adult homeless shelter, and HIV registry data, we performed sequence analysis to identify trajectories of these events and assessed their influence on engagement in HIV care and HIV viral suppression via marginal structural modeling.

**Results:**

Sequence analysis identified four trajectories; 72% of the cohort had sporadic experiences of both brief incarceration and homelessness, whereas others experienced more consistent incarceration or homelessness during early or late months. Trajectories were not associated with differential engagement in HIV care during follow-up. However, compared with PLWHA experiencing early bouts of homelessness and later minimal incarceration/homelessness events, we observed a lower prevalence of viral suppression among PLWHA with two other trajectories: those with sporadic, brief occurrences of incarceration/homelessness (0.67, 95% CI = 0.50,0.90) and those with extensive incarceration experiences (0.62, 95% CI = 0.43,0.88).

**Conclusions:**

Housing instability due to frequent jail incarceration and homelessness or extensive incarceration may exert negative influences on viral suppression. Policies and services that support housing stability should be strengthened among incarcerated and sheltered PLWHA to reduce risk of adverse health conditions.

## Introduction

Maintaining continuous engagement in HIV medical care is critical for HIV disease management for people living with HIV/AIDS (PLWHA) [1, 2]. Housing stability is considered to be an important determinant of retention in HIV medical care and for achieving and sustaining viral suppression because it helps enable PLWHA to attend regular medical visits and adhere to antiretroviral medications [3]. Housing stability may also provide other less quantifiable benefits such as stronger social networks and a sense of identity, which some researchers have postulated may motivate individuals to avoid risk behaviors and to maintain health [3]. Empirical evidence to support this relationship has been documented among PLWHA with histories of homelessness. For example, according to longitudinal data from a representative sample of New York City (NYC) PLWHA, self-reported receipt of assistance for housing problems was positively associated with appropriate HIV medical care [4]. Similarly, several studies have reported higher likelihoods of adherence to antiretroviral treatment among stably-housed PLWHA versus their homeless or unstably housed counterparts [5–7]. Along with homelessness, incarceration disrupts individuals’ housing stability and contributes to housing instability after release from incarceration. However, the association between housing stability and HIV care has rarely been described among formerly incarcerated PLWHA; current evidence is mainly concentrated on disrupted HIV care post release [8–10]. Because both events affect housing stability and incarceration is strongly correlated with homelessness, it is important to take into account dynamic aspects of incarceration events along with homelessness to more accurately characterize the impact of housing instability on continuity of HIV care. The purpose of this paper was to examine whether and how different patterns of jail incarceration and homelessness influence continuity of HIV care and HIV viral suppression among NYC PLWHA with recent experiences of both jail incarceration and homelessness, using a measure that captures sequencing and duration of jail incarceration and homelessness. Specifically, the study tested the hypothesis that frequent transitions between jail incarceration and homelessness are associated with lower likelihood of subsequent engagement in HIV care and viral suppression, plausibly due to the greater housing instability of PLWHA with this pattern.

## Materials and Methods

### Data source

The study population included 1,698 NYC adults who, prior to 2005, were diagnosed with HIV/AIDS, alive, and had spent at least one night in both NYC jail and NYC single adult homeless shelters during 2001–05. To understand dynamics between jail incarceration and homelessness, we focused on those with both types of events. They represented about 2% of NYC PLWHA during this same period. The main data source came from the combined administrative data from NYC jail and NYC single adult shelter registries, which provided jail incarceration/shelter use records and baseline demographic and criminal information. These data were then matched with the population-based NYC Department of Health and Mental Hygiene HIV/AIDS surveillance registry that contains demographic and clinical information on all diagnoses of HIV and AIDS reported in NYC, with the addition of comprehensive HIV-related laboratory reporting (including CD4 counts and viral loads test results) and deaths. This probabilistic matching process was evaluated and validation results have been published [11]. We used June 2005-June 2006 as the follow-up period for evaluating outcomes (described below) and utilized HIV laboratory test results from this time because complete viral load and CD4 test results were captured only after the New York State mandatory reporting law became effective as of June 1, 2005 [12]. Thus, to be eligible for this analysis, a PLWHA had to be alive as of January 1, 2005, which allowed us to examine HIV care outcomes during the one year follow-up time. While censoring those who died before 2005 could potentially introduce bias, this was unlikely as baseline demographic and clinical characteristics were similar between those who died during 2001–04 (n = 45) and those who survived (n = 1,698; data not shown). A small number of deaths were identified during the one year follow-up timeframe and were censored accordingly in the calculation of person-years. For deaths in 2005, 0.5 person-year was assigned (n = 64). The comparison group was comprised of NYC PLWHA who were neither incarcerated nor sheltered in a single adult shelter during 2001–05 according to an administrative database match, and were alive as of January 1, 2005 according to the NYC HIV/AIDS surveillance registry. Similar to the study population, June 2005-June 2006 was used as the follow-up period for the comparison group. The NYC Department of Health and Mental Hygiene Institutional Review Board determined that this study was not subject to institutional board review because it is not human subject research.

### Variables

Two primary outcome variables were examined in this analysis: (1) continuity of HIV medical care and (2) HIV viral suppression [2]. Continuity of HIV medical care was defined as having at least two viral load or CD4 tests during June 2005-June 2006, which were ≥90 days apart [1]. Viral suppression was defined as being achieved if there was at least one record with a viral load of <400 copies/ml during the 12-month period [2].

The exposure variable was defined using group-based trajectories of jail incarceration/homelessness during 2001–05. Analytic approaches and results of sequence analysis performed to determine these group based trajectories have been published elsewhere [13]. Potential confounders for exposure-outcome association were identified by constructing a specific Directed Acyclic Graph for this analysis [14]. These included baseline demographic characteristics (age, sex, race/ethnicity, poverty levels of neighborhoods of residency at the time of incarceration), a proxy for substance use (i.e., being charged with drug possession or transferred to substance use treatment clinics), a proxy for serious mental illness (i.e., transferred to state psychiatric hospitals or mental health treatment clinics), type of criminal charges (drug sales, violent crimes, weapon possession, public administration, property crimes, quality of life crimes, and sex crimes), age at HIV diagnosis, and development of AIDS (AIDS defining opportunistic illness or CD4 nadir of 200 cells/uL or less) [13].

### Statistical analysis

First, descriptive statistics of baseline characteristics and HIV care outcomes were summarized across trajectory groups of jail incarceration and homelessness. Bivariate association between each of these characteristics and trajectory groups was evaluated using chi-square test for categorical variables or independent t-test with Bonferroni adjustment for continuous variables. Then, marginal structural log-linear Poisson regression analysis with an offset of person-years was performed to estimate adjusted relationships between trajectories of jail incarceration/homelessness and HIV care outcomes. A stabilized inverse probability of treatment weight (IPTW) was derived from a propensity score model using trajectory groups (dependent variable) and baseline demographic, behavioral, and criminal characteristics (independent variables). Estimates from marginal structural modeling were weighted using IPTW to balance baseline characteristics across trajectory groups to improve exchangeability, ensure positivity (tightly distributed IPTW with one as a mean value), and meet stable unit treatment value assumptions (adjusting for poverty levels of neighborhood residency to address potential data dependency among some study subjects) [15–17]. A sandwich estimator for variance and a corresponding p-value were calculated because it was robust against model misspecification [16].

Multiple imputations via *IVEware* software were performed to address missing data (sex: 1% missing, race/ethnicity: 1% missing, nativity: 1% missing, poverty levels of neighborhood residency at the time of incarceration: 25% missing), which generated five imputed datasets [18]. A subset of PLWHA was then re-created and combined results of five estimates were reported according to Schafer’s approach, which accounts for within- and between-imputation variability [19].

Because the study did not collect information about behavioral and all clinical characteristics associated with retention in HIV care or viral suppression (e.g., injecting drug use), causal estimates from marginal structural modeling were potentially biased due to unobserved confounding. In a sensitivity analysis, we assessed the extent to which causal estimates might be influenced by bias due to unobserved confounding by employing VanderWeele and Arah’s bias formula derived from the relationship between outcome and unobserved confounder and that between exposure and unobserved confounder [20].

All analyses except for sequence analysis (R 2.14.2, Vienna, Austria) and imputation (*IVEware*) were performed using SAS 9.2 software (Cary, NC). Statistical significance was established if two sided p-value <0.05.

## Results

A total of 1,698 individuals living with HIV/AIDS had at least one jail incarceration event in a NYC jail and spent at least one night at a NYC single adult shelter during January 2001–May 2005. Compared with NYC PLWHA who did not experience jail incarceration or homelessness during the study period (N = 84,659), this study population was disproportionately male (84% vs. 69%) and non-Hispanic black (65% vs. 45%), and the percent of non-Hispanic whites (4% vs. 21%) and residents living in medium poverty neighborhoods (14% vs. 29%) was much smaller (Table 1). In contrast, the age distribution and stage of HIV infection were similar between the two groups.

**Table 1:** Demographic and criminal characteristics by five trajectory groups among 1,698 adults living with HIV/AIDS who were incarcerated in New York City jail and spent at least one night at New York City single adult shelter in January 2001–May 2005.

	NYC PLWHA^a^	Total	Temporary	Continuous incarceration	Continuous shelter use	Decreasing shelter use
Total	84,659	1,698	1230	317	61	90
**Exposure in 1/2001–5/2005**						
Average # of incarceration events		3	3	6	2	3
Average days in jail		139	87	369	71	85
Average jail days per incarceration		49	37	105	39	29
Average # of shelter use events		6	5	6	34	12
Average days in shelters		92	53	52	728	327
Average shelter days per shelter event		14	11	9	53	56
**Age as of 6/2002** ^b^						
18–24 years	4%	6%	6%	5%	8%	7%
25–34 years	17%	24%	24%	27%	13%	20%
35–44 years	39%	50%	49%	53%	44%	53%
45–54 years	30%	18%	18%	15%	28%	19%
55–89 years	10%	2%	3%	1%	7%	1%
**Sex** ^c^						
Male	69%	83%	83%	84%	84%	82%
Female	31%	17%	17%	16%	16%	17%
**Race/ethnicity** ^c^						
Non-Hispanic white	23%	5%	5%	3%	5%	2%
Non-Hispanic black	43%	64%	62%	68%	62%	81%
Hispanic	32%	30%	32%	27%	30%	17%
Asian	1%	0%	0%	0%	0%	0%
Others/unknown	1%	1%	1%	1%	3%	0%
**Nativity** ^c^						
United States born	62%	93%	92%	94%	90%	96%
Foreign born	13%	7%	8%	6%	10%	4%
**Poverty levels of neighborhood of residency**						
Low (<10% below poverty)	5%	2%	2%	3%	3%	2%
Medium (10 to <20%)	31%	15%	15%	16%	18%	12%
High (20 to <30%)	15%	9%	9%	10%	5%	8%
Very high (≥30%)	38%	43%	43%	44%	38%	40%
Missing	11%	31%	31%	27%	36%	38%
**Types of criminal charges**						
Drug possession		43%	40%	59%	34%	39%
Drug sales		26%	23%	36%	33%	22%
Violence		27%	24%	38%	26%	28%
Public administration		30%	28%	39%	28%	34%
Property		43%	37%	63%	38%	43%
Weapons		3%	3%	4%	3%	0%
Quality of life		7%	7%	8%	7%	7%
Sex crimes		2%	2%	2%	7%	4%
**Substance use** ^d^		46%	41%	60%	48%	48%
**Serious mental illness** ^d^		2%	2%	3%	2%	6%
**Age at HIV diagnosis**						
13–24 years	10%	10%	10%	9%	8%	8%
25–49 years	79%	84%	83%	87%	77%	86%
50+ years	11%	6%	7%	4%	15%	7%
**Stage of HIV infection at diagnosis** ^e^						
Early (HIV only)	95%	96%	96%	97%	95%	96%
Late (HIV/AIDS)	5%	4%	4%	3%	5%	4%

^a^Non-incarcerated/non-sheltered NYC residents living with HIV/AIDS.

^b^Because age was calculated at the end of year according to NYC HIV registry and raw birthdate information was not available, age of non-incarcerated/non-sheltered PLWHA was as of 12/31/2002.

^c^Because of a small % of missing data (sex: 1%, race/ethnicity: 1%, nativity: 1%) in the study cohort, sum of these numbers were not matched up with total numbers of individuals. For non-incarcerated/non-sheltered PLWHA, % of missing data were much greater than that among the study cohort.

^d^Proxy measures were used to capture substance use and prior severe mental illness conditions.

^e^Stage of HIV infection was determined according to the concurrent diagnosis where there is an AIDS diagnosis within 31 days of an HIV diagnosis.

**Fig 1:**
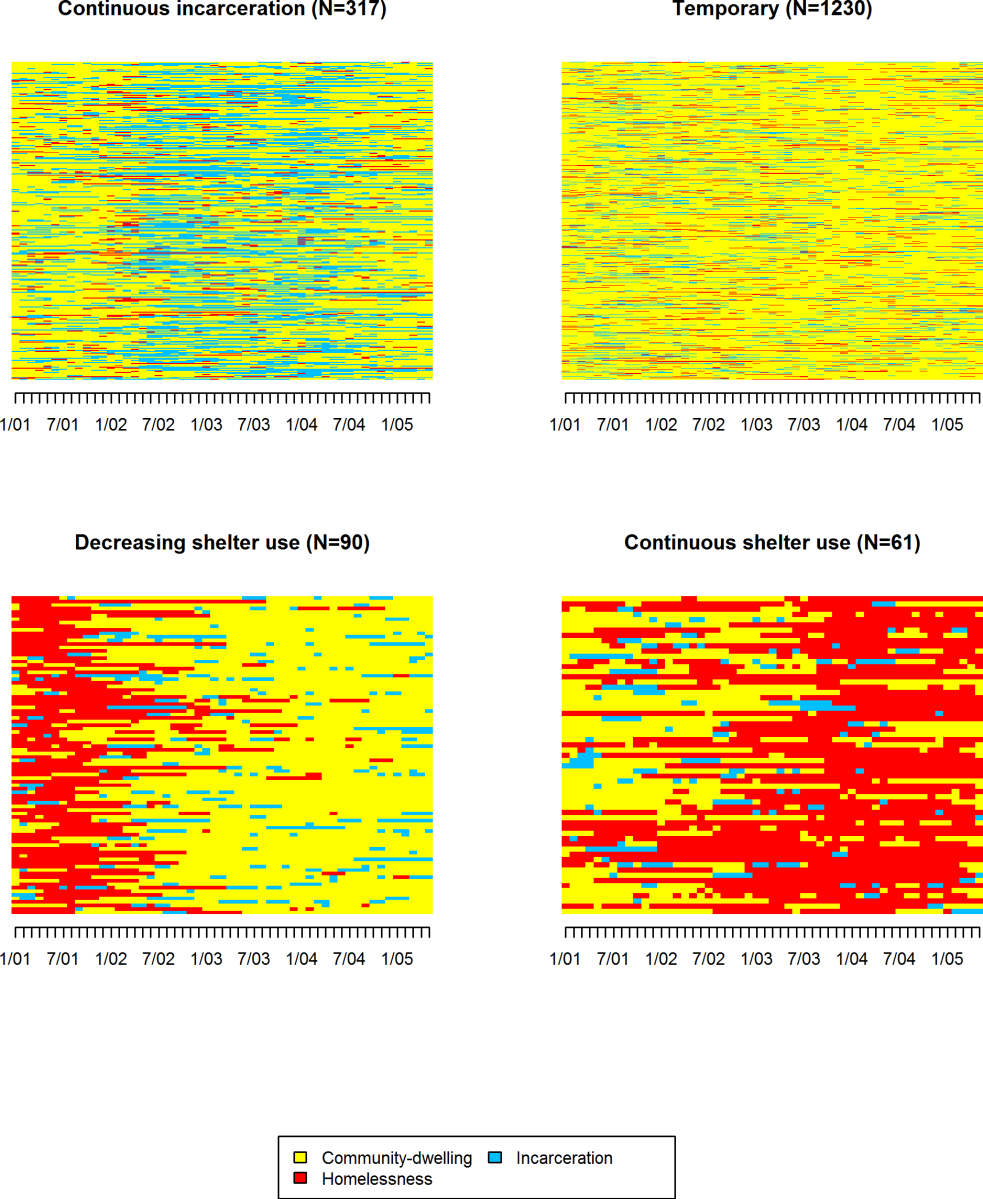
Four non-overlapping groups of jail incarceration/homelessness trajectories according to sequence analysis among 1,698 adults living with HIV/AIDS who spent at least one night in a New York City jail and at least one night at a New York City single adult homeless shelters in January 2001–May 2005. This figure describes trajectories of jail incarceration and homelessness in January 2001–May 2005. 4 trajectory groups represent distinct trajectories that were identified by sequence analysis and each individual belongs to one of 4 groups. Legend: Yellow color: Community-dwelling; Blue color: incarceration; Red color: homelessness

Fig 1 shows that among the 1,698 PLWHA experiencing both jail incarceration and homelessness from January 2001–May 2005, events were best represented by four unique trajectories. The majority (72%) had brief, intermittent jail incarceration and shelter stays during that time (n = 1,230; hereinafter *Temporary*). The second most common trajectory pattern (19%) included adults who spent extensive amounts of uninterrupted time in jail from January 2001–May 2005 (*Continuous incarceration*; n = 317). About 9% of the study cohort exhibited one of two trajectories characterized by unique shelter use patterns; one group spent extensive amounts of time in shelters (average 728 days) without much interruption, classified as having a *continuous shelter use* pattern (n = 61), and the other had continuous shelter stays during earlier months followed by periods with sporadic occurrences of brief jail incarceration/shelter stays (*Decreasing shelter use;* n = 90).

Individuals with *Continuous shelter use* pattern were older than those in other groups (Table 1). Race/ethnicity, sex, nativity, and neighborhood poverty were unrelated to trajectory group. Across all trajectory groups, approximately half of the study cohort had an indication of substance use, while prevalence of serious mental illness was highest in the *Decreasing shelter use* group. Individuals charged with drug possession, drug sales, property, and violent crimes were more likely to exhibit the *Continuous incarceration* trajectory compared to those with other criminal charges. Age at HIV diagnoses and likelihood of progression to AIDS were similar across trajectory groups.

Among the study cohort, 50% had at least two HIV-related laboratory tests for viral load or CD4 count, which were ≥90 days apart during one-year follow-up time (Table 2). This was similar among non-incarcerated/non-sheltered NYC PLWHA, 50% of whom also experienced at least two HIV lab tests in the same period. Prevalence of engagement in HIV care was roughly similar across all trajectory groups, ranging from 41% to 53%. During June 2005-June 2006, 29% of the study cohort experienced viral suppression; prevalence of viral suppression was highest among individuals who stayed in shelters during the early months and dwelled in the community afterwards (*Decreasing shelter use*). During the same period, 41% of non-incarcerated/non-sheltered NYC PLWHA experienced viral suppression.

**Table 2:** Percentages of continuity of HIV care and HIV viral suppression^a^ by trajectory groups among adults living with HIV/AIDS with recent experiences of jai incarceration and homelessness, New York City, June 2005-June 2006.

	N	% of continuity of HIV care^a^	% of viral suppression^a^
Non-incarcerated/non-sheltered NYC PLWHA	84,659^b^	51%	41%
The study population	1,698	50%	29%
Temporary	1230	53%	29%
Continuous incarceration	317	41%	26%
Continuous shelter use	61	48%	31%
Decreasing shelter use	90	49%	41%

^a^Continuity of HIV care was defined as having at least two viral load or CD4 tests during June 2005-June 2006, which were ≥90 days apart. Viral suppression was defined as being achieved if there was at least one record with a viral load of <400 copies/ml during the 12-month period.

^b^Non-incarcerated/non-sheltered NYC PLWHA who were diagnosed and alive before 2005.

Results from marginal structural regression analysis showed that trajectories of jail incarceration and homelessness were not significantly associated with subsequent engagement in HIV care during June 2005-June 2006 (Table 3). Among NYC PLWHA with recent histories of jail incarceration and homelessness, those with *Temporary* relative to *Decreasing shelter use* patterns had 0.67 times lower prevalence of viral suppression during the same one-year period (95% Confidence Interval (CI) = 0.50, 0.90). In addition, those with *Continuous incarceration* pattern were less likely to experience viral suppression, compared with those with *Decreasing shelter use* pattern (PR = 0.62, 95% CI = 0.43, 0.88).

**Table 3:** Prevalence ratio for continuity of HIV care and HIV viral suppression by trajectory groups among 1,698 adults living with HIV/AIDS with recent experiences of jai incarceration and homelessness, New York City, June 2005-June 2006.

Trajectory groups	Continuity of HIV care		HIV viral suppression	
	**PR** ^a^ ^,^ ^b^	**95% CI** ^b^	**PR** ^a^ ^,^ ^b^	**95% CI** ^b^
Temporary	1.04	0.82, 1.32	**0.67**	**0.50, 0.90**
Continuous incarceration	0.81	0.61, 1.07	**0.62**	**0.43, 0.88**
Continuous shelter use	0.76	0.49, 1.17	0.71	0.52, 1.22
Decreasing shelter use	Reference		Reference	

CI, confidence interval; PR, prevalence ratio.

^**a**^Prevalence ratio was estimated from log-linear Poisson models. Numbers in bold indicate statistical significance at p <0.05.

^b^Inverse probability of treatment weight was used to control for bias due to confounding. Covariates for the model for continuity of HIV care included age, sex, race/ethnicity, nativity, neighborhood poverty, a proxy measure of substance use, a proxy measure of mental illness, criminal charges due to drug sales, violent crimes, weapon possession, public administration, property crimes, quality of life crimes, sex crimes, ages of HIV diagnoses, and stage of HIV infection. The same sets of covariates except for criminal charges due to weapon possession were used for the model for viral suppression.

Results from the sensitivity analysis showed how the upper bound of 95% CI for the prevalence ratio for viral suppression could be affected by adjusting for unobserved confounding (see S1 Fig). As long as the association between unobserved behaviors and viral suppression was greater than 1 and particular behaviors (e.g., injecting drug use) among individuals with *Temporary* relative to *Decreasing shelter use* patterns were more prevalent (δ >1), the estimated prevalence ratio would have remained significant. On the other hand, if particular behaviors among individuals with *Temporary* relative to *Decreasing shelter use* patterns were 20% less prevalent (δ = 0.8) and association between these unobserved behaviors and viral suppression was strong (PR ≥ 2.5 or γ ≥ 2.5), the estimated prevalence ratio would not have been statistically significant.

## Discussion

In this study, almost half of NYC adults living with HIV/AIDS who were sheltered and incarcerated from January 2001–May 2005 experienced inadequate engagement in HIV care during the one-year follow-up period. This percentage was similar between the study cohort and non-incarcerated/non-sheltered NYC PLWHA who were diagnosed and alive prior to 2005. Unlike with measures of HIV care engagement, however, overall prevalence of viral suppression was much lower among the study cohort relative to non-incarcerated/non-sheltered NYC PLWHA, suggesting that treatment initiation and/or adherence to treatment might be more difficult among PLWHA with exposure to jail incarceration and homelessness.

Within this population of NYC PLWHA who experienced jail incarceration and homelessness, those who sporadically experienced such events or spent extensive amounts of time in jail from January 2001–May 2005 were less likely to experience viral suppression during the one-year follow-up period compared with those who left homeless shelters during early months and rarely returned. However, there was no difference in engagement in HIV care by trajectory group.

The null association between trajectories of jail incarceration/homelessness and engagement in HIV care did not support the hypothesis that frequent transitions of short-term jail incarceration and homelessness are associated with poor engagement in HIV primary care. Prevalence of HIV care engagement (51%), regardless of trajectory group, was similar to that among non-incarcerated/non-sheltered NYC PLWHA (50%). It was higher than that among youth and adults living with HIV/AIDS in 13 United States jurisdictions (45%) [1], and than what was observed for formerly-incarcerated PLWHA in 10 United States urban areas who were followed for 6 months after release from jail (38%) [21]. Our finding implies that those with recent histories of incarceration and homeless events were able to receive regular HIV care. Since affordable HIV medical services and various social services are widely available in NYC via safety net programs such as the Ryan White program, and are promoted in NYC neighborhoods, jails, and homeless shelters, experiencing life disruptions due to jail incarceration and homelessness might not act as a barrier to subsequent continuity of HIV care for those already connected to care [12].

Unlike engagement in care, prevalence of viral suppression significantly differed by trajectories of jail incarceration and homelessness. This implies that initiation of and/or adherence to treatment might be more difficult among PLWHA with sporadic, often frequent, exposure to brief jail incarceration and homelessness or those with extensive incarceration experiences, compared with those who had shelter stays only during the early portion of the study period with rare return to shelters or incarcerations subsequently. Given that permanent housing assistance and services were available to NYC PLWHA who qualified, this finding may reflect better access to services such as case management among the reference group that resulted in their being started on treatment and/or assisted them in adhering to HIV treatment regimens. Another possible mechanism is that being persistently out of shelter and jail is indicative of housing stability. Housing stability and the associated likelihood of medication adherence may influence some provider decisions about whether or not initiate HIV treatment [3].

Adults experiencing the *Continuous incarceration* pattern, as opposed to *Decreasing shelter use* pattern, were also less likely to achieve viral suppression, whereas there was no significant difference between those with *Continuous shelter use* pattern and the reference group. This difference between the two continuous patterns may suggest that incarcerated NYC PLWHA experience more difficulty being connected to housing services and experiencing housing stability compared with those in shelters during the early 2000s, which could negatively impact initiation of and/or adherence to HIV treatment regimens [22]. Additionally, because those with continuous incarceration experiences were more likely to be transferred from jails to prisons and clinical data from prisons were missing, viral suppression status could have been misclassified (and the proportion virally suppressed underestimated). This limitation, along with post-release challenges of stable housing, might partly explain why low viral suppression was observed among those with extensive jail incarceration, unlike previous studies that reported positive impacts of incarceration on viral suppression during incarceration [23,24]. Additional data on housing, case services, and prison data are needed to fully understand the mechanism that particular trajectories were associated with viral suppression, while engagement in HIV care was independent of trajectories of incarceration/homelessness.

The study finding supports our conclusion from previous work that excess risk of HIV-related mortality associated with the *Temporary* pattern could be a manifestation of disruptions to HIV treatment due to transitions between jails and shelters [13]. In the current study, viral suppression may act as a mediator of the *Temporary* pattern-HIV-related mortality association. Sporadic experiences of brief jail incarceration and homelessness, as opposed to undisrupted community-dwelling, may contribute to elevating HIV-related mortality risk among NYC PLWHA with recent incarceration and homelessness by creating environments where essential HIV care and adherence cannot be initiated and/or gets disrupted. Future studies that link mortality data with data form jail and shelter registries and the HIV surveillance registry are warranted to further explore and test mechanisms, such as declining CD4 counts, which may contribute to excess HIV-related mortality associated with a pattern of sporadic exposure to brief jail incarceration and homelessness.

This study has some limitations. First, there were limited data on demographic, behavioral, and clinical characteristics of PLWHA who were both incarcerated and sheltered. Despite use of proxy measures of mental illness and substance use, we might not have been able to completely capture PLWHA with these conditions. Individuals with *Temporary* pattern may be different in systematic ways that may be causally related to risk for not initiating HIV treatment and/or HIV treatment non-adherence than those with *Decreasing shelter use* pattern. Depending on the direction of unobserved confounding between these patterns, the relationship between viral suppression and *Temporary* pattern could be biased either toward or away from the null, as seen in the sensitivity analysis. For example, suppose that 50% of individuals with *Temporary* pattern had no documented history of injecting drug use. If the percent of non-injecting drug users among *Decreasing shelter use* individuals was 63% and non-injecting drug users were 2.5 times or higher more likely to experience viral suppression, *Temporary* versus *Decreasing shelter use* pattern would have been no longer significantly associated with viral suppression. Second, data about housing quality and types of housing services were not collected. Even though homeless PLWHA in NYC have a legal right to be housed, being out of shelter and jail does not necessarily mean being stably housed in the community, and some types of housing assistance are more likely to facilitate stability than others. Third, we considered PLWHA who did not have viral load test results during the analytic period to be virally unsuppressed [25]. Because test results from state prisons could not be included in this analysis and PLWHA with *Continuous incarceration* pattern were more likely to move to state prisons than those with other patterns, selection bias could not be ruled out. Fourth, data about antiretroviral treatment were not available for analyses. Without knowing length of time under the treatment, understanding an impact of housing stability on viral suppression was limited. Lastly, because antiretroviral treatment was not recommended for all PLWHA and recommendations were only tied to low CD4 count rather than high viral loads during the study period, lack of viral suppression might not serve as a proxy for lack of care of poor adherence. Although stage of HIV infection at diagnosis was controlled for, drawing a linkage between viral suppression and HIV treatment should be made with caution.

Despite these limitations, a main strength of the study is the use of a measure that captured dynamic aspects of jail incarceration and homelessness, potentially reflecting differing levels of housing instability over time. In addition, use of IPTW allowed for explicitly testing causal assumptions, further strengthening internal validity of the finding. Lastly, administrative data provided almost complete coverage of HIV-related outcome ascertainment among PLWHA in NYC.

In conclusion, this study suggests that life disruption due to jail incarceration and homelessness does not appear to introduce additional barriers to receiving HIV care at recommended intervals but may exert negative influences on achieving and maintaining viral suppression, which is associated with poor clinical outcomes, survival, and onward HIV transmission. It also highlights the relatively better outcomes among the reference group (i.e., higher prevalence of viral suppression) who might experience stable community-dwelling for a longer period. This finding provides important evidence for policies and services (e.g., case management) in support of housing stability among incarcerated and sheltered PLWHA in order to reduce risk of adverse health conditions.

## Supporting Information

S1 Fig Estimated upper bound of 95% confidence interval of prevalence ratio for HIV viral suppression by the Temporary pattern adjusted for an unobserved confounder (*U*) among 1,698 PLWHA with jail incarceration and homelessness, New York City, June 2005-June 2006. This figure illustrates the results from the sensitivity analysis. Lines represent varying estimates of upper bound of 95% confidence interval of prevalence ratio for HIV viral suppression by *Temporary* pattern over *Decreasing shelter use* pattern, depending on varying prevalence estimates of unobserved confounder and association between HIV viral suppression and unobserved confounder.
(DOCX)
