# Supplementary material for: Influence of Jail Incarceration and Homelessness Patterns on Engagement in HIV Care and HIV Viral Suppression among New York City Adults Living with HIV/AIDS
Source: PLoS One. 2015 Nov 23;10(11):e0141912. doi: 10.1371/journal.pone.0141912 (PMC4657891; doi:10.1371/journal.pone.0141912)
Supplement: S1 Fig — This figure illustrates the results from the sensitivity analysis. Lines represent varying estimates of upper bound of 95% confidence interval of prevalence ratio for HIV viral suppression by Temporary pattern over Decreasing shelter use pattern, depending on varying prevalence estimates of unobserved confounder and association between HIV viral suppression and unobserved confounder. (DOCX) [file pone.0141912.s001.docx]

Estimated upper bound of 95% confidence interval of prevalence ratio for HIV viral suppression by the Temporary pattern adjusted for an unobserved confounder (*U*) among 1,698 PLWHA with jail incarceration and homelessness, New York City, June 2005-June 2006

CI, confidence interval; PR, prevalence ratio.

Notes: the upper bound of 95% CI of prevalence ratio of viral suppression by *Temporary* pattern was 0.90 from Table 3. *P(U=1|a=1,x)* was arbitrarily set as 0.5.

*U* = unmeasured confounder; *δ* = *P(U=1|a=1, x) / P(U=1|a=0,x)*; *γ* = *PR* of HIV viral suppression by *U*.
